# Comment on: "Postoperative evaluation of interleukin-8 and C1q/TNF-related protein-12 in patients undergoing coronary artery bypass grafting surgery"

**DOI:** 10.1590/1806-9282.20250435

**Published:** 2025-07-07

**Authors:** Ömer Faruk Rahman, Fevzi Ayyıldız

**Affiliations:** 1 İzmir Bakırçay University, Department of Cardiovascular Surgery – İzmir, Turkey.; 2 Aydın Adnan Menderes University, Department of Cardiovascular Surgery – Aydın, Turkey.

Dear Editor,

We found the study by Sisakht et al., titled "*Postoperative evaluation of interleukin-8 and C1q/TNF-related protein-12 in patients undergoing coronary artery bypass graft surgery*" to be noteworthy and intriguing^1^. However, we believe that certain aspects of the study require further discussion.

The study appears to have limited consideration of preoperative patient variables that could influence interleukin (IL)-8 levels. Atrial fibrillation is a well-known comorbidity associated with inflammation, and its role in the pathogenesis of atrial fibrillation has been demonstrated in several studies^2,3^. Therefore, the absence of atrial fibrillation in the preoperative variable table may have an impact on the results. Similarly, chronic obstructive pulmonary disease (COPD) and hyperlipidemia, which are also linked to inflammation, were not included as preoperative variables in this study, which could be considered a limitation.

Furthermore, as mentioned in the article, coronary artery bypass grafting (CABG) is a procedure that triggers inflammation through various mechanisms and contributes to postoperative adverse events^4^. The absence of important intraoperative variables related to CABG, such as cross-clamp time and cardiopulmonary bypass duration, can be considered a limitation of this study.

In the postoperative intensive care period, certain variables may influence the inflammatory process and IL-8 levels. One of these is red blood cell transfusion, which has been reported to increase IL-8 levels^5^. Including red blood cell transfusion data in the study could have facilitated a more accurate interpretation of the results. Additionally, variables such as intra-aortic balloon pump use and intensive care unit length of stay are other parameters that could be associated with the inflammatory process and may have influenced the study outcomes.

Finally, we would like to express our appreciation to Sisakht et al. for their valuable study. We believe that a future study incorporating the aforementioned data could provide more objective insights into the inflammatory process.

## Data Availability

The datasets generated and/or analyzed during the current study are available from the corresponding author upon reasonable request.

## References

[1] Sisakht MT, Pourghadamyari H, Dehesh T, Karim ZR, Nazari-Robati M (2025). Postoperative evaluation of interleukin-8 and C1q/TNF-related protein-12 in patients undergoing coronary artery bypass graft surgery. Rev Assoc Med Bras (1992).

[2] Melenovsky V, Lip GY (2008). Interleukin-8 and atrial fibrillation. Europace.

[3] Rafaqat S, Rafaqat S, Rafaqat S (2022). Major interleukins: role in the pathogenesis of atrial fibrillation. J Cardiac Arrhythmias.

[4] Aljassim O, Karlsson M, Wiklund L, Jeppsson A, Olsson P, Berglin E (2006). Inflammatory response and platelet activation after off-pump coronary artery bypass surgery. Scand Cardiovasc J.

[5] Johnson JL, Moore EE, Gonzalez RJ, Fedel N, Partrick DA, Silliman CC (2003). Alteration of the postinjury hyperinflammatory response by means of resuscitation with a red cell substitute. J Trauma.

